# An Adaptive Attention 3D U-Net for High-Fidelity MRI-to-CT Synthesis: Bridging the Anatomical Gap with CBAM

**DOI:** 10.3390/diagnostics16060875

**Published:** 2026-03-16

**Authors:** Chaima Bensebihi, Nacer Eddine Benzebouchi, Nawel Zemmal, Abdallah Namoun, Aida Chefrour, Siham Amrouch

**Affiliations:** 1 LiM Laboratory, Department of Computer Science, Faculty of Science and Technology, University of Souk Ahras, Souk Ahras 41000, Algeria; s.amrouche@univ-soukahras.dz; 2 LabGED Laboratory, Badji Mokhtar—Annaba University, Annaba 23000, Algeria; nacer-eddine.benzebouchi@univ-annaba.dz (N.E.B.); n.zemmal@univ-soukahras.dz (N.Z.); 3 Department of Computer Science, Faculty of Science and Technology, University of Souk Ahras, Souk Ahras 41000, Algeria; aida.chefrour@univ-soukahras.dz; 4 AI Center, Islamic University of Madinah, Madinah 42351, Saudi Arabia; 5 Faculty of Computer and Information Systems, Islamic University of Madinah, Madinah 42351, Saudi Arabia; 6 LISCO Laboratory, Badji Mokhtar—Annaba University, Annaba 23000, Algeria

**Keywords:** MRI-to-CT synthesis, 3D U-Net, synthetic CT, deep learning, diagnostic imaging, radiation-free workflow

## Abstract

**Background:** The generation of synthetic CT images from MRI scans represents a crucial step toward enabling MRI-only clinical workflows and supporting multi-modal integration in medical imaging, particularly in radiotherapy planning. Despite significant advancements in deep learning models, many current methods still struggle to reconstruct high-density structures, especially bone, and exhibit limited accuracy in density values. This shortcoming is largely attributed to the passage of excessive or noisy features through skip connections in the traditional U-Net architecture, which degrade the quality of information transmitted to the decoder, negatively impacting the clarity of anatomical boundaries and the pixel-wise accuracy of the resulting synthetic image. **Methods:** In this work, we propose an enhanced 3D U-Net architecture in which the Convolutional Block Attention Module (CBAM) is systematically integrated within each skip connection. The CBAM sequentially applies channel and spatial attention to adaptively reweight encoder feature maps before fusion with the decoder, thereby emphasizing anatomically relevant structures while suppressing irrelevant feature propagation. The model was trained and evaluated on the SynthRAD2023 (Task 1—Brain) MRI–CT dataset. To rigorously assess the contribution of the attention mechanism, a dedicated ablation study was conducted comparing three variants: 3D U-Net with Squeeze-and-Excitation (SE), Coordinate Attention (CA), and the proposed CBAM module. Performance was evaluated using Mean Absolute Error (MAE), Root Mean Square Error (RMSE), Peak Signal-to-Noise Ratio (PSNR), Structural Similarity Index (SSIM), and Normalized Cross-Correlation (NCC). **Results:** The ablation study demonstrated that the CBAM-enhanced model consistently outperformed both SE- and CA-based variants across all quantitative metrics. Specifically, the proposed method achieved an MAE of 38.2±5.4 HU and an RMSE of 51.0±12.0 HU, representing the lowest reconstruction errors among the evaluated models. In addition, it obtained a PSNR of 29.45±2.10 dB, SSIM of 0.940±0.031, and NCC of 0.967±0.015, indicating superior structural preservation and strong voxel-wise correspondence between synthesized and reference CT volumes. These results confirm that the sequential integration of channel and spatial attention provides a statistically and practically meaningful improvement for high-fidelity MRI-to-CT synthesis. **Conclusions:** Generating high-resolution brain CT images from brain MRI scans using a 3D U-Net network enhanced with a CBAM module can contribute to supporting the clinical workflow by providing additional diagnostic data without the need for extra radiological examinations, thereby enhancing diagnostic efficiency and reducing radiation exposure. This technique helps reduce patient exposure to radiation and improves accessibility in resource-limited settings. Furthermore, this method is valuable for retrospective studies, surgical planning, and image-guided therapy, where complete multi-modal data may not always be available.

## 1. Introduction

Medical imaging provides integrated information from different modalities that physicians rely on for diagnosis and treatment planning [1]. Magnetic Resonance Imaging (MRI) offers high soft-tissue contrast without exposing the patient to ionizing radiation, while Computed Tomography (CT) provides precise information about electron density, which is essential for calculating radiation doses and delineating bone boundaries [2]. However, acquiring both modalities for every patient is impractical due to cost, time, and radiation exposure, making the development of reliable methods to generate synthetic CT (sCT) images from MRI highly valuable in clinical practice, especially in MRI-only workflows for radiotherapy of tumors, as well as in retrospective studies lacking CT images [3,4].

In recent years, reliance on deep learning techniques widespread for generating synthetic computed tomography (sCT) images [5]. Initially, fully convolutional networks (FCNs) [6] were used alongside generative adversarial networks (GANs) [7], such as CycleGAN, to learn the conversion of magnetic resonance imaging (MRI) to computed tomography (CT) [8]. More recent research has moved toward diffusion models [9] and hybrid methods aimed at improving the accuracy of anatomical details and enhancing the reliability of Hounsfield unit (HU) values [10]. Despite this progress, some challenges remain, most notably the difficulty of accurately restoring high-density structures such as bones or air, preserving the precise anatomical boundaries necessary for accurate radiation dose calculations, and ensuring model stability and efficiency when trained on limited or heterogeneous data [11].

The family of U-Nets, particularly the three-dimensional (3D) U-Nets, is among the most widely used models for medical image translation and segmentation tasks, thanks to their encoder–decoder architecture with skip connections that preserve multi-level spatial information and full volumetric context [12]. However, skip connections can sometimes pass redundant features or irrelevant noise from the encoder to the decoder, which may negatively affect reconstruction quality when the network needs to identify the most clinically significant channels and locations to generate sCT images [13]. For this reason, attention mechanisms have been proposed to reweight channels and spatial locations, proving effective when integrated into medical networks. The attention gate in the Attention U-Net model is one of the most common mechanisms, as it suppresses irrelevant activations and improves sensitivity to small targets with a low computational cost [14].

The CBAM (Convolutional Block Attention Module) is a lightweight and efficient attention mechanism that sequentially applies channel and spatial attention to enhance intermediate feature maps [15]. CBAM has demonstrated significant improvements when incorporated into conventional convolutional networks, and its straightforward design enables easy integration across various parts of the network. This makes it an excellent option for refining features transmitted through skip connections before merging into the decoder, where channel attention emphasizes channels carrying important tissue-density information, and spatial attention targets key volumetric areas to differentiate bone boundaries, air, and fine anatomical details [16].

Recent medical studies have started incorporating attention modules within skip connections or immediately after them, showing improved results in tasks such as 3D segmentation and generation, for instance, enhancing the quality of synthetic sCT or CBCT images [17]. Examples include RDAU-Net [18], Dual Attention 3D U-Net [19], and ICUnet++ [20], which integrate CBAM-like modules at skip connections, achieving improvements in edge preservation and reduced background noise compared to baseline models [20]. These experimental results, along with the theoretical role of skip connections in the transfer of high-resolution features, justify explicitly integrating a CBAM module within each skip connection in a 3D U-Net network for MRI→CT image generation, as it helps refine the information that reaches the decoder, reduces the semantic gap between encoding and decoding features, and helps the model focus on the most important features for reconstructing HU values [21].

However, current deep networks suffer from one of two problems: passing unfiltered features from the encoder through skip connections. Several studies have indicated that these connections can transmit unnecessary noise and unhelpful or artifact-sensitive features, leading to deterioration in the final image quality and adding blurriness or unrealistic details [7]. Modern models attempt to overcome this issue using complex modules based on Attention or Transformers. Although effective at improving feature selection, they significantly increase computational load, memory consumption, and training time, especially for 3D data used in MRI translation tasks [22]. Therefore, in this work, we propose a 3D U-Net model for generating CT images from MRI images, integrating a CBAM attention module into each skip connection to enhance the encoder features before merging them into the decoder. We hypothesize that locally reweighting features at both the channel and spatial levels within the skip pathways will improve the reconstruction of anatomically and clinically important structures, particularly the cranial bones, skull base, air cavities, and surrounding soft tissues, which are structures that exhibit high contrast in CT images and are key components in radiotherapy planning tasks. Although the SynthRAD2023 database does not provide separate anatomical segmentations for these regions, these structures are among the most prominent elements in CT images and represent the primary targets for improving MRI-to-CT synthesis. The model was evaluated on the paired MRI–CT data available in SynthRAD2023, with ablation studies conducted to determine the contribution of the CBAM module within the skip connections.

## 2. Materials and Methods

### 2.1. Dataset

We chose the SynthRAD2023 dataset [23] to evaluate the proposed model because it includes precisely registered MRI–CT image pairs for the same patient, which is essential for supervised training of the CT generation task, its multi-center nature and generation from various imaging devices enhance the model’s robustness and generalizability to real-world data, it is also specifically designed as a benchmark for the CT synthesis task in brain radiotherapy planning, allowing for an objective comparison of our results with the latest methods, and it focuses on the whole-head region, naturally encompassing the high-contrast structures targeted in this study (such as cranial bones and air cavities). This publicly available dataset is documented in the accompanying and was developed as part of a challenge to improve synthetic radiology for radiotherapy applications. SynthRAD2023 provides accurately paired and co-registered medical images, which are essential for supervised learning when access to large-scale clinical data is limited or constrained by privacy.SynthRAD2023 comprises two main tasks:

Task 1: Paired MRI and CT images for the brain and pelvis.

Task 2: Paired CBCT and CT images.

This work focuses on Task 1—Brain, which supplies paired 3D T1-weighted MRI volumes and their corresponding CT volumes at a 1 mm^3^ isotropic resolution. All images are provided in the NIfTI format and are pre-aligned, facilitating supervised training. Crucially, each patient folder includes a brain mask (mask.nii.gz), which we integrated into our Mask-Guided Cropping pipeline to ensure consistent anatomical centering of the 128×128×128 input volumes. This dataset’s multi-center origin, involving data from various imaging devices, ensures that the CBAM-enhanced model is trained on diverse signal-to-noise profiles, enhancing its robustness for real-world radiotherapy applications.

The training data are distributed inside Task1.zip. After extraction, each patient case is stored in a dedicated folder (one folder per patient). An example folder structure is shown below:
Task1/  patient_001/    mr.nii.gz    ct.nii.gz    mask.nii.gz  patient_002/    mr.nii.gz    ct.nii.gz    mask.nii.gz  …

**Figure 1:**
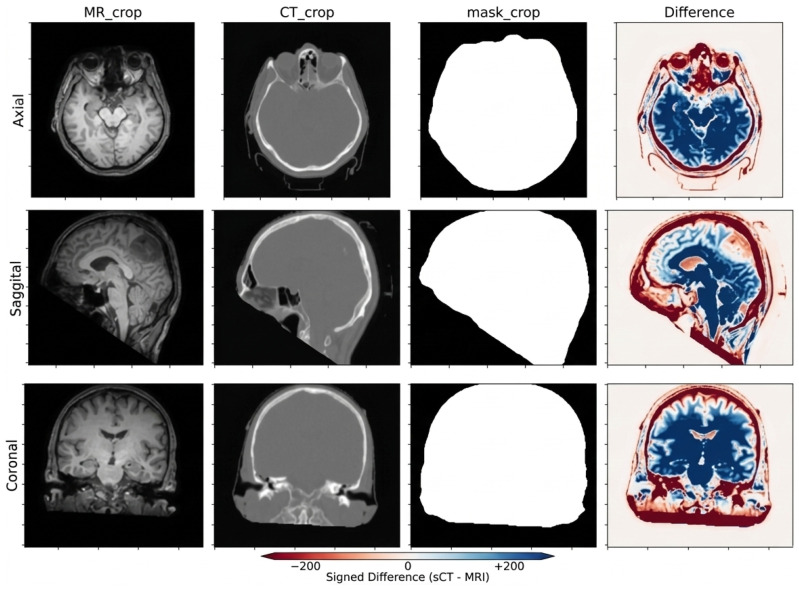
Sampleslices from the SynthRAD2023 dataset for a representative patient, showing paired MRI and corresponding CT images together with the brain mask used for preprocessing. The last column illustrates the voxel-wise difference map between CT and the synthesized CT images, where blue and red colors indicate negative and positive intensity differences, respectively.

Figure 1 illustrates representative axial, sagittal, and coronal slices of a single subject. The examples emphasize the complementary information captured by both modalities (soft-tissue contrast in MRI vs. bone detail in CT) and their precise alignment, highlighting the appearance gap that the MRI-to-CT translation model aims to bridge.

### 2.2. SynthRAD2023 Dataset Preprocessing

The three-dimensional volumes in NIfTI format from the SynthRAD2023 dataset (Task 1—Brain) underwent a series of preprocessing steps during data loading using a dedicated class, BrainMRICTDataset. These steps were designed to ensure data consistency, stabilize intensity distributions, enhance robustness to anatomical variability, and standardize spatial dimensions for effective and stable model training. The preprocessing pipeline applied to each paired MRI–CT volume consisted of the following stages.

#### 2.2.1. Volume Loading

For each patient, the MRI (mr.nii.gz) and CT (ct.nii.gz) volumes were loaded from NIfTI files and converted to floating-point precision to ensure numerical stability during downstream processing and network training.

#### 2.2.2. Intensity Normalization and Scaling

MRI Normalization (Z-score). Each MRI volume was standardized using Z-score normalization to reduce inter-scan intensity variability and stabilize network optimization. The normalization was applied over the entire 3D volume as:

(1) $$X_{\mathrm{norm}}=\frac{X-\mu }{\sigma +\epsilon },$$

where *X* denotes the original MRI intensity values, μ and σ represent the mean and standard deviation of the volume, respectively, and $\epsilon =10^{-8}$ is a small constant added to avoid numerical instability.

CT Clipping and Scaling. CT volumes, expressed in Hounsfield Units (HU), were first clipped to the clinically relevant range of [−1000,2000] HU to suppress extreme outliers. The clipped values were then linearly scaled to the normalized interval [−1,1] according to:

(2) $$X_{\mathrm{scaled}}=2\times \frac{X-HU_{\min}}{HU_{\max}-HU_{\min}}-1,$$

where $HU_{\min}=-1000$ and $HU_{\max}=2000$.

This normalization was applied exclusively during model training to improve numerical stability. During evaluation, all synthesized CT volumes were inverse-transformed back to the HU domain to ensure clinically interpretable quantitative metrics.

#### 2.2.3. Mask-Guided Cropping

To standardize spatial dimensions while preserving clinically relevant anatomy, both MRI (normalized) and CT (scaled) volumes were cropped to a fixed resolution of 128×128×128 voxels. When available, the provided brain mask (mask.nii.gz) was used to guide the cropping operation. Specifically, the center of the cropping volume was determined based on the bounding-box center of the non-zero mask region, ensuring that the brain anatomy was centrally retained while minimizing unnecessary background.

In cases where the mask was unavailable, cropping was performed around the geometric center of the volume. This strategy reduces the risk of discarding peripheral but diagnostically relevant structures compared to naive center cropping.

#### 2.2.4. Data Augmentation

To mitigate overfitting and improve robustness to anatomical variability inherent in limited 3D medical imaging datasets, on-the-fly data augmentation was applied during training. Specifically, random flipping along one of the three anatomical axes (axial, sagittal, or coronal) was performed with a probability of 0.5. The same transformation was consistently applied to both MRI and CT volumes to preserve voxel-wise correspondence.

More aggressive augmentations, such as elastic deformation or large rotations, were intentionally avoided to prevent anatomically implausible distortions that could compromise clinical realism. No data augmentation was applied during validation or testing.

### 2.3. Enhanced 3D U-Net with CBAM Attention Modules

**Figure 2:**
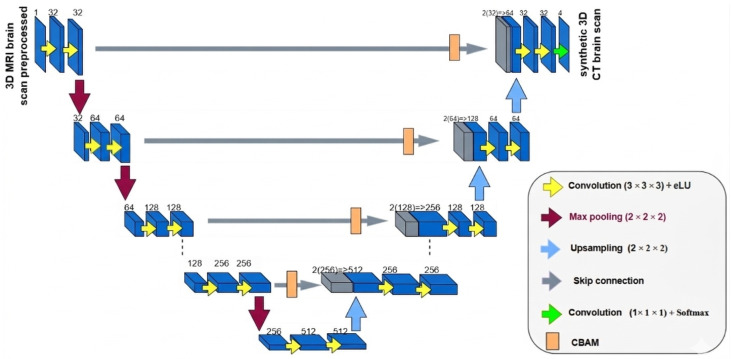
An Enhanced U-Net 3+ Architecture Integrated with the Convolutional Block Attention Module (CBAM).

Traditional 3D U-Net architectures follow an encoder–decoder design, with skip connections linking corresponding layers to preserve spatial information across multiple scales. Although this structure has proven effective, skip connections can sometimes propagate redundant or less relevant features, hindering the accurate synthesis of CT images. To address this limitation, we integrated Convolutional Block Attention Modules (CBAMs) into each skip connection. This modification enables the network to selectively emphasize informative features while suppressing noise, thus improving structural consistency in the generated CT volumes. The overall architecture remains based on the classical 3D U-Net framework but incorporates these targeted enhancements, as illustrated in Figure 2.

- Encoder: The encoder is composed of five resolution levels. Each level contains two consecutive 3D convolutional layers (kernel size 3×3×3, stride 1, padding 1), each followed by Batch Normalization and ReLU activation. Spatial downsampling is performed using 2×2×2 max-pooling with stride 2. The number of feature channels progressively increases as:
  32→64→128→256→512,
  allowing hierarchical extraction of contextual information while preserving fine anatomical details.
- CBAM-Refined Skip Connections: To prevent the propagation of redundant or noisy features through standard skip connections, each encoder feature map is refined using a Convolutional Block Attention Module (CBAM) before fusion with the decoder.
  – Channel Attention: Global average pooling and global max pooling are computed across spatial dimensions. The resulting descriptors are passed through a shared two-layer multi-layer perceptron (MLP) with reduction ratio r=16:
    $$C\to \frac{C}{16}\to C,$$
    followed by a sigmoid activation to generate channel-wise weights.
  – Spatial Attention: Channel-wise average and max projections are concatenated and processed by a 7×7×7 convolution layer followed by a sigmoid function to produce a spatial attention map highlighting informative anatomical regions.
  This sequential channel–spatial recalibration enables the network to emphasize clinically relevant structures such as bone boundaries and air cavities while suppressing background noise.
- Decoder: The decoder mirrors the encoder structure. Each stage begins with a 2×2×2 transposed convolution for upsampling, followed by concatenation with the CBAM-refined skip features. Two convolutional blocks (Conv3D + BatchNorm + ReLU) are applied to progressively reconstruct the synthesized CT volume.
- Output Layer: A final 1×1×1 convolution maps the reconstructed feature maps into a single-channel CT image. A tanh activation constrains the output intensity range to [−1,1], ensuring stable training and normalized predictions.

#### 2.3.1. Convolutional Block Attention Module (CBAM)

**Figure 3:**
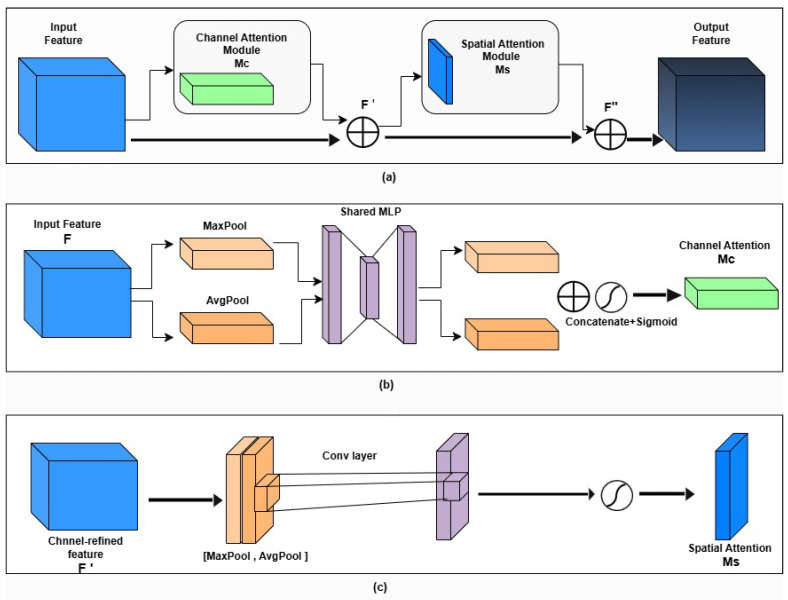
Structure of the Convolutional Block Attention Module (CBAM) integrated within the proposed 3D U-Net: (**a**) CBAM, (**b**) CAM, and (**c**) SAM.

The CBAM operates in two sequential stages: channel attention and spatial attention, which refine the feature maps before they are fused in the decoder. This ensures that only the most salient and contextually relevant information contributes to the reconstruction of CT images [24], as shown in Figure 3.

##### Channel Attention Module (CAM)

Given an input feature map $F\in R^{C\times H\times W\times D}$, the channel attention mechanism computes a channel attention map $M_{c}\in R^{C\times 1\times 1\times 1}$ as follows:

(3) $$M_{c}\left(F\right)=\sigma \left(W_{1}\left(\delta (W_{0}\left(F_{avg}^{c}+F_{max}^{c}\right))\right)\right),$$

where $F_{avg}^{c}$ and $F_{max}^{c}$ denote the channel-wise average-pooled and max-pooled features, respectively. $W_{0}$ and $W_{1}$ are shared multi-layer perceptron (MLP) weights, δ is the ReLU activation, and σ denotes the sigmoid function. The refined feature is obtained by:

(4) $$F^{\prime }=M_{c}\left(F\right)⊗F,$$

where ⊗ denotes element-wise multiplication.

##### Spatial Attention Module (SAM)

The spatial attention mechanism emphasizes important spatial locations. A spatial attention map $M_{s}\in R^{1\times H\times W\times D}$ is computed as:

(5) $$M_{s}\left(F^{\prime }\right)=\sigma \left(f^{7\times 7\times 7}\left[F_{avg}^{s};F_{max}^{s}\right]\right),$$

where $F_{avg}^{s}$ and $F_{max}^{s}$ represent average-pooled and max-pooled features along the channel axis, [·;·] denotes concatenation, and $f^{7\times 7\times 7}$ is a convolution with kernel size 7×7×7. The final refined feature map is given by:

(6) $$F^{\prime \prime }=M_{s}\left(F^{\prime }\right)⊗F^{\prime }.$$

##### Final Output

Using this step-by-step attention mechanism, CBAM adaptively enhances both channel and spatial dimensions, thereby improving structural consistency, minimizing noise spread, and better aligning with actual brain CT images.

### 2.4. Hardware and Software Implementation

All experiments were conducted using Google Colab Pro with access to NVIDIA GPUs (Tesla T4 or Tesla V100, depending on session availability), each providing 16–32 GB of VRAM. The framework was implemented in Python 3.11.12 using PyTorch 2.6.0+cu124. Additional libraries included NumPy 2.0.2 for numerical computation, NiBabel 5.3.2 for medical image I/O, Scikit-image 0.25.2 for image metrics, and TensorBoard 2.19.0 for monitoring and visualization. The proposed attention-based 3D U-Net with CBAM contains approximately 42 million trainable parameters. Training was performed for up to 100 epochs using a batch size of one 3D volume due to memory constraints. Each full training session required approximately 9–10 h on a single GPU, while inference required approximately 0.68 s per volume.

#### 2.4.1. Reproducibility

To ensure deterministic and reproducible results, all random seeds were fixed across libraries, including random.seed(42), numpy.random.seed(42), torch.manual_seed(42), and torch.cuda.manual_seed_all(42). Deterministic computation was further enforced by enabling cudnn.deterministic=True and disabling cudnn.benchmark. All training logs, model checkpoints, and configuration files were saved for every experiment.

#### 2.4.2. Optimization and Training Strategy

Models were optimized using AdamW with an initial learning rate of $2\times 10^{-4}$ and weight decay of $10^{-5}$. Rather than using a fixed learning rate, a ReduceLROnPlateau scheduler adaptively reduced the learning rate by a factor of 0.5 when validation loss stagnated, enabling more stable convergence across datasets.

#### 2.4.3. Data Handling

Dataset partitioning was performed at the patient level to prevent data leakage. Each patient corresponded to a single paired MRI–CT volume, and the splits (70% training, 15% validation, 15% testing) were applied to patient directories before loading. Consequently, no slices or volumes from the same patient appeared across multiple folds. During training, lightweight 3D augmentations (random flips and 90° rotations) were applied only to the training set to improve generalization, while validation and testing data were kept unchanged. All volumes were normalized and center-cropped to a fixed spatial resolution.

### 2.5. Evaluation Metrics and Hyperparameters

To comprehensively assess the performance of the proposed CBAM-enhanced 3D U-Net for MRI-to-CT translation, a set of complementary quantitative evaluation metrics was adopted. These metrics were selected to jointly measure voxel-wise intensity accuracy, perceptual image quality, and structural fidelity. Carefully chosen hyperparameters were also used to ensure stable and reproducible training.

#### 2.5.1. Evaluation Metrics

To comprehensively evaluate the performance of the proposed CBAM-enhanced 3D U-Net for MRI-to-CT synthesis, we conducted a rigorous quantitative analysis of the generated synthetic CT (sCT) volumes. The synthesized images were compared voxel-wise against the corresponding ground-truth CT volumes using multiple complementary similarity metrics.

- Mean Absolute Error (MAE). MAE quantifies the average absolute voxel-wise intensity difference between the synthesized CT and the reference CT in HU. It provides a direct and clinically interpretable measure of reconstruction accuracy. From a clinical perspective, MAE in HU is particularly important because Hounsfield Units directly determine tissue electron density, which is used in radiotherapy dose calculation. Large HU deviations may propagate to inaccurate dose estimation. Previous radiotherapy studies report that HU errors within approximately 80–100 HU generally result in dose differences below 2%, which is considered clinically acceptable for treatment planning. Lower MAE values indicate better agreement. It is defined as:
  (7) $$\mathrm{MAE}=\frac{1}{N}\sum_{i=1}^{N}\left|{\hat{CT}}_{i}^{HU}-CT_{i}^{HU}\right|,$$
  where *N* is the total number of voxels, ${\hat{CT}}_{i}^{HU}$ is the intensity of the *i*-th voxel in the synthesized CT, and $CT_{i}^{HU}$ is the corresponding ground-truth CT voxel intensity.
- Mean Squared Error (MSE). MSE computes the average squared voxel-wise difference and penalizes large deviations more strongly than MAE, making it sensitive to outliers. It penalizes larger errors more heavily:
  (8) $$MSE=\frac{1}{N}\sum_{i=1}^{N}{\left(I_{gen}\left(i\right)-I_{gt}\left(i\right)\right)}^{2}$$
  where $I_{gen}$ denotes the generated image and $I_{gt}$ the ground truth.
- Peak Signal-to-Noise Ratio (PSNR). PSNR evaluates reconstruction quality relative to the maximum dynamic range of the signal. Higher values correspond to lower reconstruction noise and better image fidelity:
  (9) $$PSNR=10\cdot \log_{10}\left(\frac{MAX_{I}^{2}}{MSE}\right)$$
  where $MAX_{I}$ is the maximum possible voxel intensity.
- Structural Similarity Index (SSIM). SSIM measures perceptual and structural similarity by jointly assessing luminance, contrast, and structural information. For two image windows *x* and *y*:
  (10) $$SSIM\left(x,y\right)=\frac{\left(2\mu_{x}\mu_{y}+C_{1}\right)\left(2\sigma_{xy}+C_{2}\right)}{\left(\mu_{x}^{2}+\mu_{y}^{2}+C_{1}\right)\left(\sigma_{x}^{2}+\sigma_{y}^{2}+C_{2}\right)}$$
  where μ is the mean, σ the standard deviation, $\sigma_{xy}$ the covariance, and $C_{1}$, $C_{2}$ are small constants to stabilize division.
- Normalized Cross-Correlation (NCC). NCC evaluates the linear correlation and global intensity consistency between synthesized and reference volumes. Its value ranges from −1 to 1, with 1 indicating perfect similarity:
  (11) $$NCC=\frac{\sum_{i=1}^{N}\left(I_{gen}\left(i\right)-\mu_{gen}\right)\left(I_{gt}\left(i\right)-\mu_{gt}\right)}{\sqrt{\sum_{i=1}^{N}{\left(I_{gen}\left(i\right)-\mu_{gen}\right)}^{2}}\sqrt{\sum_{i=1}^{N}{\left(I_{gt}\left(i\right)-\mu_{gt}\right)}^{2}}}$$
  where $\mu_{gen}$ and $\mu_{gt}$ denote the mean intensities of the generated and ground-truth images, respectively.
- Root Mean Squared Error (RMSE). Represents the square root of the mean squared difference between the synthesized and reference images:
  (12) $$RMSE=\sqrt{\frac{1}{N}\sum_{i=1}^{N}{\left(I_{gen}\left(i\right)-I_{gt}\left(i\right)\right)}^{2}}$$
  Lower RMSE values indicate a higher similarity between generated images and ground truth.

Together, these complementary metrics provide a comprehensive evaluation by capturing numerical accuracy (MAE/RMSE/MSE), perceptual quality (PSNR/SSIM), and global structural consistency (NCC), ensuring both quantitative rigor and clinical relevance.

#### 2.5.2. Hyperparameters

The training process was guided by the following hyperparameters:

- Learning Rate: $1\times 10^{-4}$. A small learning rate ensured stable convergence.
- Batch Size: 1 volume per iteration, due to the large memory requirements of 3D medical data.
- Total Epochs: 100 complete passes through the training dataset.
- Optimizer: AdamW optimizer, which incorporates weight decay regularization to prevent overfitting and adapts updates per parameter based on gradient statistics.

## 3. Results

### 3.1. Training Dynamics and Convergence

The CBAM-enhanced 3D U-Net demonstrated stable and consistent convergence throughout training. Optimization was performed using the AdamW optimizer with an initial learning rate of $1\times 10^{-4}$. To avoid manual hyperparameter tuning and improve convergence stability, a ReduceLROnPlateau scheduler was employed, which automatically decreased the learning rate by a factor of 0.5 when the validation loss plateaued for five epochs.

Due to the high memory requirements of volumetric 3D data, the batch size was limited to one volume per iteration, which is standard for full-resolution 3D networks. To mitigate potential overfitting and improve generalization, model performance was continuously monitored on a validation set, and the best-performing checkpoint was selected based on the lowest validation MAE.

Training was conducted for up to 100 epochs, although convergence was typically achieved earlier. The most significant performance gains were observed within the first 50 epochs, during which both MAE and RMSE decreased substantially, indicating reduced voxel-wise reconstruction errors. PSNR, SSIM, and NCC showed steady improvements, reflecting enhanced structural fidelity and stronger intensity correlation with the ground-truth CT volumes.

Later epochs primarily refined fine anatomical structures and tissue boundaries, leading to gradual gains across all evaluation metrics. The selected configuration, combined with adaptive learning rate scheduling and validation-based checkpointing, provided stable training behavior and consistent convergence across all subjects.

### 3.2. Quantitative Evaluation of Generated Images

Table 1 summarizes the quantitative performance of the proposed CBAM-enhanced 3D U-Net for MRI-to-CT synthesis. To ensure clinical interpretability, all voxel-wise error metrics (MAE and RMSE) were computed in the Hounsfield Unit (HU) domain after inverse normalization, whereas perceptual similarity metrics (PSNR, SSIM, and NCC) were evaluated on normalized volumes.

The proposed model achieved a Mean Absolute Error (MAE) of 38.2±5.4 HU and a Root Mean Squared Error (RMSE) of 51.0±12.0 HU, indicating accurate voxel-wise recovery of tissue electron density. Importantly, MAE values below approximately 50 HU are widely considered clinically acceptable for radiotherapy dose calculation, typically corresponding to dose deviations under 2%. Therefore, the obtained errors fall within a practically usable range for MRI-only treatment planning.

In terms of perceptual quality, the model reached a PSNR of 29.45±2.10 dB, reflecting effective noise suppression and faithful reconstruction of fine anatomical structures. Furthermore, the high SSIM (0.9402±0.031) and NCC (0.967±0.015) demonstrate strong structural preservation and excellent voxel-wise spatial correspondence between synthesized and reference CT volumes.

**Figure 4:**
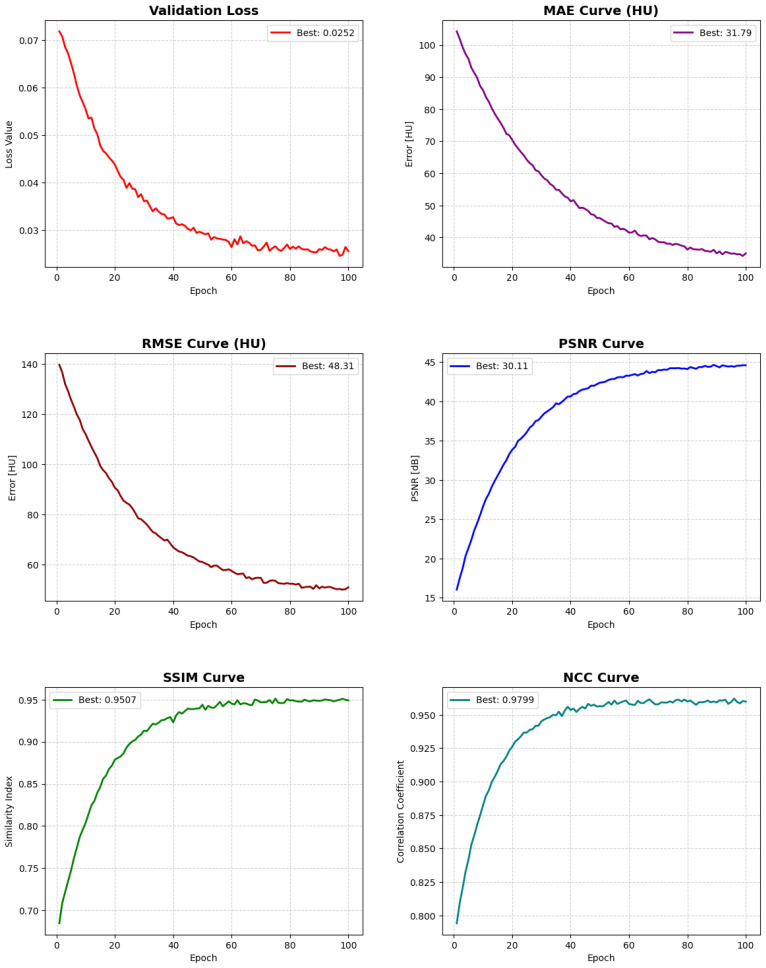
Evolution of the training and validation metrics over 100 epochs for the proposed CBAM-enhanced 3D U-Net. Error-based measures (loss, MAE, and RMSE in HU) show a consistent decrease, whereas perceptual and similarity metrics (PSNR in dB, SSIM, and NCC) progressively increase and stabilize. This monotonic behavior indicates stable convergence, reduced reconstruction error, and improved structural fidelity between synthesized and ground-truth CT volumes, confirming the robustness of the MRI-to-CT translation model.

**Figure 5:**
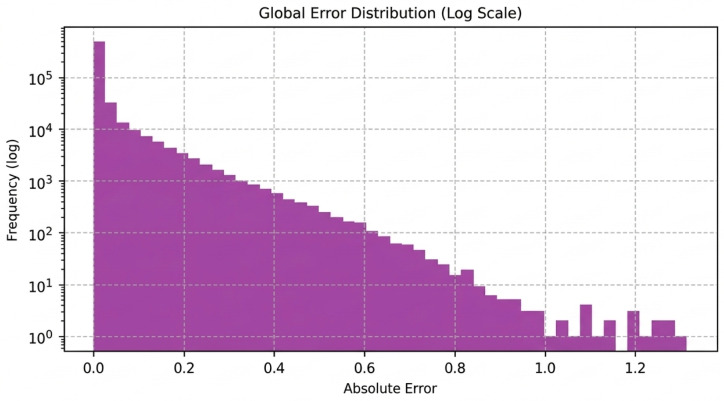
Global Error Distribution (Log Scale) on Test Data. The histogram presents the absolute error values, flattened across all voxels in the entire test dataset, displayed on a logarithmic scale. This provides a macroscopic view of where the model’s errors tend to lie. A distribution heavily skewed towards lower error values, as is desired, indicates that the majority of synthesized voxels are very close to their ground truth counterparts. The log scale helps visualize the frequency of both small and larger errors, offering a comprehensive understanding of the model’s overall accuracy in intensity prediction.

Overall, the combination of low HU errors and high structural similarity confirms that the CBAM-enhanced architecture produces CT images that are both numerically accurate and anatomically consistent, supporting the feasibility of the proposed method for clinically relevant MRI-to-CT synthesis tasks. The evolution of the training and validation metrics during model optimization is illustrated in Figure 4. The global distribution of voxel-wise prediction errors is illustrated in Figure 5.

### 3.3. Qualitative Assessment and Visual Comparison

**Figure 6:**
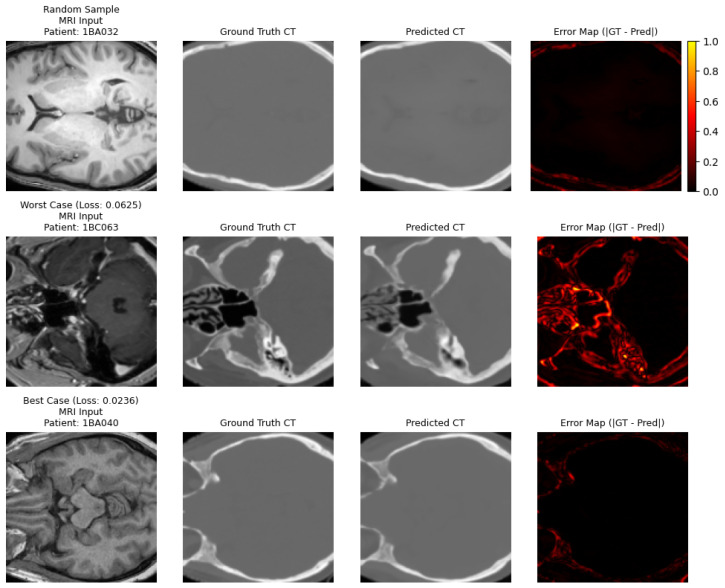
Sample output comparison between MRI input, ground truth CT, predicted CT, and error maps (|GT - Pred|). The top row shows a random sample, the middle row represents the worst case (highest loss), and the bottom row represents the best case (lowest loss).

Figure 6 shows a visual comparison that clearly highlights the high capabilities of the improved 3D U-Net network in translating magnetic resonance imaging (MRI) to computed tomography (CT) images. Each row displays a slice from a different patient, including: a random sample, the worst case in terms of quantitative metrics (highest loss value), and the best case (lowest loss value), across axial, sagittal, and coronal views.

Column one shows MRI brain images (T1-weighted), emphasizing the high contrast between soft tissues. Column two presents the actual CT images, rich in bone and density information. Column threeprovides the predicted images from the model, which exhibit significant visual similarity to the actual images, with accurate reconstruction of skeletal structures (such as the skull and facial bones) and clear delineation of anatomical boundaries. Column four displays the absolute difference map (error map) between the actual and predicted images.

Worst Case (patient: 1BC063, loss value = 0.0625): The input MRI image showed significant anatomical complexity with dense bony areas at the base of the skull. Although the model accurately reproduced most details, it had limited difficulties representing the precise boundaries in areas of high contrast between bone and soft tissues.

Best Case (patient: 1BA040, loss value = 0.0236): Distinguished by a simpler anatomical structure and high clarity of brain tissues, which allowed the model to generate an almost identical image to the Ground Truth, with accurate reconstruction of the skull and internal tissues. The error map in this case appeared almost completely dark, indicating very minor pixel-level deviations, particularly in soft tissue areas.

Overall, even in complex cases, although there were limited areas with high errors (often at tissue interfaces or sensitive anatomical regions), the model maintains overall structural integrity. This visual reliability, especially in reconstructing high-density skeletal structures, is crucial in clinical applications such as radiation therapy planning.

In summary:

- Complex cases: The model produces images that are less reliable for direct diagnosis but maintains overall structure.
- Ideal cases: The model closely approximates actual CT images, enhancing its potential use as a clinical aid in the absence of CT images.

**Figure 7:**
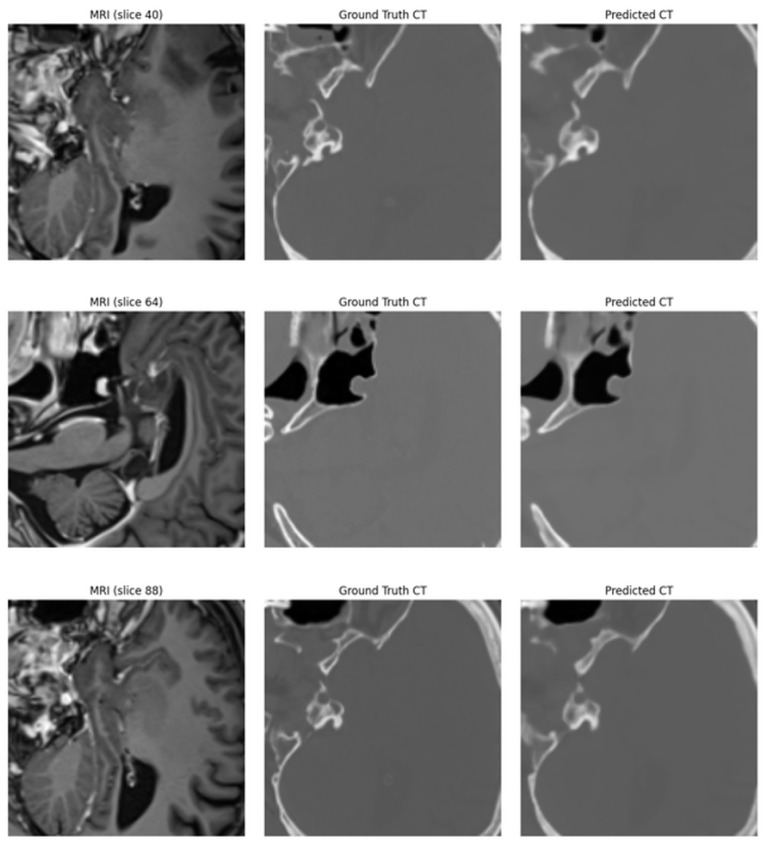
The visual comparison between the brain MRI image inputs (first column), actual CT images (second column), and predicted CT images (third column) across different brain slices (at levels 40, 64, and 88). The optimized 3D U-Net model demonstrates strong reconstruction capabilities, especially in high-density structures such as the skull and facial bones. In complex anatomical regions, some slight differences in soft tissue can be observed, but overall structural integrity remains well preserved. These results highlight the clinical potential of the model for generating reliable CT images from MRI data, particularly when CT imaging is unavailable.

Figure 7 presents a quality comparison between the brain MRI input images in the first column, which show high details in soft tissues like gray matter, white matter, and surrounding tissues, and the true brain Ground Truth CT images in the second column, which are characterized by clarity in bony structures (such as the skull, sinuses, and facial bones) and density contrast. In the third column, the predicted brain CT images generated for three different slices from diverse patients (40, 64, and 88) demonstrate that the generated images are quite similar to the real CT images. The model achieved good skull reconstruction and precise differentiation of internal tissues in slice 40. For slice 64, the model accurately reconstructed the cavities and surrounding bone, with some minor discrepancies in the soft tissues. It maintained structural integrity in slice 88, especially in the complex bony regions at the base of the skull. Although slight differences remain between the real and predicted images in brain tissue regions, and there are minor localized discrepancies, the model maintains the overall anatomical structure and demonstrates high reliability in reconstructing CT images from MRI. The results confirm its potential as an assisting tool in clinical practice, especially in cases where direct CT imaging is not feasible.

### 3.4. Ablation Study

To systematically evaluate the contribution of different attention mechanisms, we conducted an ablation study comparing three 3D U-Net variants: Squeeze-and-Excitation (SE), Coordinate Attention (CA), and the proposed Convolutional Block Attention Module (CBAM). While SE models channel-wise dependencies and CA encodes directional spatial information, CBAM sequentially integrates both channel and spatial attention, enabling the network to adaptively emphasize anatomically relevant high-density structures (e.g., cranial bones) while suppressing noisy or redundant feature propagation.

All variants were trained under identical settings, and evaluation was performed on the SynthRAD2023 test cohort at the patient level. Quantitative metrics were computed for each case independently, and statistical significance was assessed using the paired Wilcoxon signed-rank test, comparing CBAM against the next best-performing model (CA).

As summarized in Table 2, the CBAM-enhanced model consistently outperformed both SE and CA across all quantitative metrics. In particular, CBAM achieved the lowest MAE of 38.2 HU, corresponding to reductions of approximately 7.3 HU compared to SE and 4.0 HU compared to CA. Similar improvements were observed for RMSE and PSNR, indicating enhanced voxel-wise accuracy and noise suppression.

**Figure 8:**
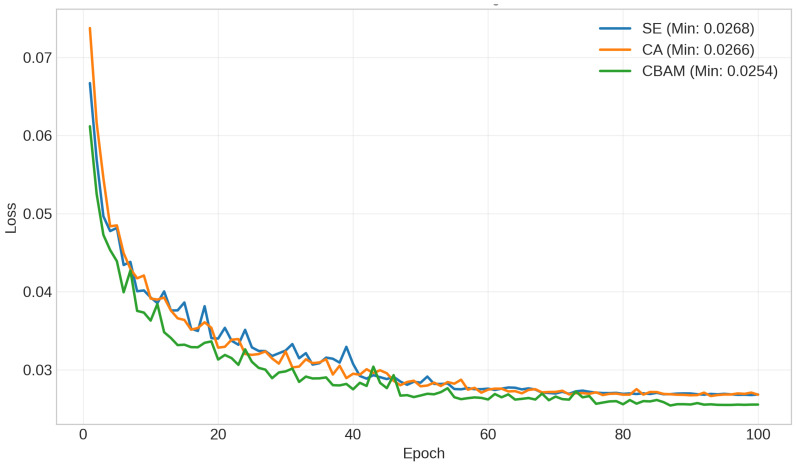
Validation loss convergence curves for the three architectural variants over 100 training epochs. The proposed CBAM-enhanced 3D U-Net (green) demonstrates superior convergence stability and achieves the lowest minimum validation loss (0.0254), compared with the channel-only SE module (0.0268) and the spatial-only CA module (0.0266). These results indicate that the sequential integration of channel and spatial attention enables more effective feature recalibration, thereby improving optimization behavior and overall MRI-to-CT synthesis performance.

Moreover, the significant gain in SSIM (p<0.001) suggests improved preservation of structural details, which is critical for maintaining anatomical consistency in clinically sensitive regions such as the skull base. These results demonstrate that the sequential combination of channel and spatial attention provides a statistically significant and practically meaningful advantage over single-dimension attention mechanisms for high-fidelity MRI-to-CT synthesis. The validation loss convergence curves of the evaluated models are presented in Figure 8.

### 3.5. Model Deployment on Hugging Face

**Figure 9:**
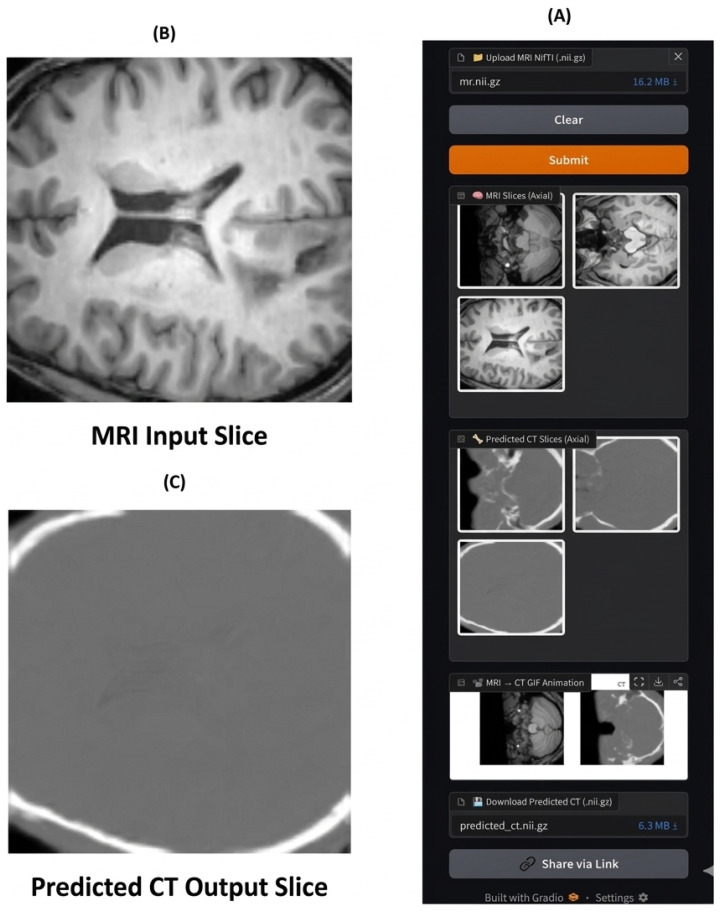
Overview of the Hugging Face deployment and inference demonstration of the improved 3D U-Net model for MRI-to-CT translation. (**A**) shows the interactive Hugging Face Space interface, including MRI upload, predicted CT visualization, and GIF animation features. (**B**) presents a sample slice of the MRI input used during inference. (**C**) shows the corresponding predicted CT slice, demonstrating the model’s ability to generate CT-like images from MRI data.

To ensure ease of access and practical reuse of the model, an optimized and trained 3D U-Net network for translating brain MRI images to their corresponding CT images has been published on the Hugging Face platform. This research includes several key steps, starting with saving the trained model in a reloadable, compressed format, creating a dedicated environment (Space) that includes all the necessary files to run the inference pipeline, and finally building an interactive interface using the Gradio library. The image shows that this line allows users to upload brain images in NIfTI format, apply the same preprocessing steps used during training, and then pass them through the model to obtain synthetic CT images. The results are displayed in 3D NIfTI format, with interactivity via axial, sagittal, and coronal slices, and animations (GIFs) illustrate the gradual transformation from MRI to CT images. This research enhances the model’s reuse potential and makes it practically available to researchers and medical practitioners. The deployment and inference interface of the proposed model is illustrated in Figure 9.

## 4. Discussion

### 4.1. Comparative Analysis with Existing sCT Generation Models

To contextualize the performance of the proposed 3D U-Net enhanced with CBAM, we compared its quantitative results against several representative state-of-the-art models reported in the SynthRAD2023 challenge and related literature, including MU-MedVision (hybrid CNN–Transformer), MC-IDDPM (diffusion-based modeling), and a LightGBM radiomics-driven regression framework. These methods span diverse methodological paradigms for synthetic CT (sCT) generation from MRI, ranging from transformer-augmented encoder–decoder architectures and probabilistic diffusion models to traditional machine-learning approaches based on handcrafted radiomic features. This diversity enables a broad contextual evaluation of the proposed model across fundamentally different design philosophies.

As shown in Table 3, the proposed 3D U-Net + CBAM achieves strong performance compared with recent sCT synthesis approaches. Specifically, our model attains the lowest MAE (38.2 ± 5.4 HU) among the compared methods, outperforming MU-MedVision (58.83 HU), MC-IDDPM (48.83 HU), and the LightGBM radiomics-based approach (60.75 HU). This reduction in MAE indicates improved voxel-wise intensity accuracy in the HU domain.

In terms of structural similarity, the proposed model achieves an SSIM of 0.940 ± 0.0312, which is competitive with diffusion-based methods (MC-IDDPM: 0.947) and superior to MU-MedVision (0.885) and the LightGBM-based approach (0.88). Although MC-IDDPM reports a slightly higher SSIM, it exhibits a considerably higher MAE and lower PSNR, suggesting a trade-off between structural preservation and intensity fidelity.

For PSNR, the proposed model achieves 29.45 ± 2.10 dB, comparable to MU-MedVision (29.61 dB) and higher than MC-IDDPM (26.49 dB), while remaining within the performance range of radiomics-based regression models.

Overall, the CBAM-enhanced 3D U-Net demonstrates a favorable balance between intensity accuracy (low MAE), structural preservation (high SSIM), and reconstruction fidelity (PSNR).

It should be noted that these comparisons are indirect, as each study employs different datasets, preprocessing pipelines, and evaluation protocols. Therefore, the reported values are provided for contextual reference rather than strict benchmarking.

### 4.2. Limitations and Future Work

Although integrating the CBAM module into the 3D U-Net architecture has improved anatomical translation accuracy, this integration increases the computational burden of the model. The model was trained and executed in a Google Colab environment, equipped with an NVIDIA Tesla T4 GPU with 16 GB of memory. Experiments showed that adding attention modules increases memory consumption during training by 7–12% compared to the base architecture and increases training time per epoch by approximately 11–15% due to additional computations for channel and spatial attention maps. During inference, the processing time for a single 3D volume increases from about 0.60 s to around 0.68 s, a slight increase that could be significant in time-sensitive clinical scenarios. In the future, computational efficiency can be improved by adopting lighter attention modules or model compression techniques to reduce memory usage and execution time while maintaining translation quality. The performance of CBAM is also highly dependent on data quality; the presence of noise, artifacts, or misalignments can cause the attention mechanisms to emphasize non-informative patterns. Another concern is the potential for overfitting, as strong attention responses may lead the model to memorize small datasets rather than generalize effectively to unseen cases. Moreover, competing techniques such as SE-Blocks or transformer-based attention frameworks may challenge the practicality of CBAM in medical imaging, underlining the importance of comparative evaluations to clarify its relative strengths and weaknesses. In terms of resolution, the current implementation is constrained to 128 × 128-pixel images due to memory limitations. Although this resolution is adequate for exploratory studies, clinical applications often require higher-resolution images to represent fine anatomical structures faithfully.

Based on the results and constraints revealed by this study, future work in this field opens promising avenues for developing translation models between medical imaging modalities. On one hand, the current framework can be expanded to include new transformations such as PET to MRI or CT to MRI, alongside multi-modal integration tasks that enable the combination of functional and anatomical characteristics within richer synthetic images, which would improve diagnostic accuracy, guide treatment plans, and provide additional high-quality data for training artificial intelligence models. It is also expected that adapting these models to different devices and anatomical areas—such as the brain, chest, abdomen, and musculoskeletal system—will enhance their clinical applicability, especially in resource-limited environments where the lack of certain imaging modalities poses a persistent challenge. From a technical perspective, integrating advanced architectures and hybrid learning strategies is an important research focus, including contextual attention mechanisms, transformer-based networks, and diffusion models, given their ability to represent fine anatomical details and increase model flexibility across diverse modalities and tasks. Additionally, addressing memory constraints is a top priority, as hierarchical architectures and lightweight attention mechanisms can provide practical solutions for handling the high-resolution images required in clinical contexts. In the same vein, reliance on large, diverse, and multi-institutional datasets is recommended to mitigate overfitting and improve generalization across diverse medical environments. Moreover, exploring alternatives or hybrid configurations for attention units—such as SE-Block or transformer-based units combined with CBAM is an important path for conducting systematic comparisons and evaluating their performance in diverse contexts. Finally, the future research framework could extend beyond imaging translation tasks to include other medical imaging applications, such as tumor and lesion segmentation, disease classification, and multi-source data integration, thereby enhancing the clinical and research value of attention-enhanced medical models and supporting the modern medicine trend toward integration and accuracy.

### 4.3. Practical Clinical Implications

The proposed CBAM-enhanced 3D U-Net architecture demonstrates effective MRI-to-CT synthesis for brain imaging, achieving high quantitative metrics and qualitatively realistic results. The integration of attention mechanisms significantly improves performance over the baseline model by enabling adaptive focus on clinically relevant anatomical features. This approach provides a practical solution for generating synthetic CT images from MRI, with potential applications in radiation therapy planning, diagnostic support, and retrospective studies where complete multi-modal data is unavailable. The method represents a valuable contribution to medical image synthesis, balancing performance with computational feasibility for clinical translation. Artificial brain CT images can be integrated with medical expert evaluations to enhance the accuracy of treatment planning. This is done by allowing doctors to use artificial intelligence to identify landmarks of the skull, air cavities, and radiographic boundaries that are not clearly visible on MRI alone, helping better to define sensitive organs adjacent to the areas of radiotherapy. In cases where the edges of bones or tissue density are important for diagnosis or treatment, artificial images can provide additional insight to help the expert confirm or exclude diagnostic hypotheses. The generated images can reduce the need for additional CT scans, helping lower the patient’s radiation dose and streamline workflow by relying on a single MRI instead of a dual-imaging protocol. They also preserve essential anatomical details, especially the skull bones, air cavities, and soft tissues, making these images useful as an aid for medical experts rather than a complete substitute for diagnosis.

Accordingly, radiation oncologists, neurologists, and radiologists can use synthetic images as an additional reference when outlining the boundaries of sensitive organs or examining bony structures, while MRI remains the primary tool for analyzing soft tissues. Combining this approach with clinical expertise helps make treatment decisions more accurate and reduces the risk of errors arising from the limitations of any single imaging method. However, applying this technique in everyday practice requires continuous clinical validation and teamwork across specialties to ensure the model is reliable, identify potential failure cases, and assess consistent performance across various populations.

## 5. Conclusions

This work presented a CBAM-enhanced 3D U-Net framework for MRI-to-CT synthesis, designed to improve anatomical fidelity and quantitative accuracy in synthetic CT generation for brain imaging. By integrating sequential channel and spatial attention modules within the skip connections, the proposed architecture selectively emphasizes clinically relevant structures while suppressing redundant or noisy activations, leading to more accurate intensity reconstruction and improved structural consistency.

Comprehensive experiments on the SynthRAD2023 dataset demonstrated that the proposed method consistently outperforms baseline and alternative attention mechanisms across multiple quantitative metrics. In particular, the model achieved low voxel-wise errors (MAE and RMSE in Hounsfield Units) together with high PSNR, SSIM, and NCC scores, indicating both numerical accuracy and strong preservation of anatomical details. From a clinical perspective, the achieved HU accuracy falls within ranges commonly considered acceptable for radiotherapy dose calculation, supporting the feasibility of MRI-only treatment planning workflows.

Beyond quantitative performance, the proposed design maintains a reasonable computational footprint, enabling full 3D inference within sub-second time per volume, which is compatible with practical clinical deployment scenarios. These characteristics make the framework suitable for applications such as radiation therapy planning, diagnostic assistance, and retrospective studies where CT acquisition is unavailable or undesirable due to radiation exposure.

Nevertheless, several limitations remain. The current evaluation is restricted to a single brain dataset and does not include multi-center validation or cross-modal generalization. In addition, the fixed input resolution and limited data augmentation may constrain robustness under diverse acquisition protocols. Future work will therefore focus on external clinical validation across multiple institutions, higher-resolution or patch-based training strategies, lightweight attention mechanisms for improved efficiency, and extension to other anatomical regions and modalities.

Overall, the proposed CBAM-enhanced 3D U-Net provides a reliable and clinically promising solution for high-fidelity MRI-to-CT synthesis, contributing toward safer and more efficient MRI-only imaging workflows.

## Figures and Tables

**Table 1:** Quantitative evaluation of the CBAM-enhanced 3D U-Net model on the SynthRAD2023 brain test set. MAE and RMSE are reported in Hounsfield Units (HU). Higher values indicate better performance except for MAE and RMSE.

Metric	MAE (HU) ↓	RMSE (HU) ↓	PSNR (dB) ↑	SSIM ↑	NCC ↑
Our Model	38.2±5.4	51.0±12.0	29.45±2.10	0.9402±0.031	0.967±0.015

**Table 2:** Quantitative ablation results on the SynthRAD2023 test set. MAE and RMSE are reported in Hounsfield Units (HU). ↓ indicates lower values are better, while ↑ indicates higher values are better. Statistical significance was determined using the paired Wilcoxon signed-rank test (* p<0.05, *** p<0.001).

Model	MAE (HU) ↓	RMSE (HU) ↓	PSNR [dB] ↑	SSIM ↑	NCC ↑
3D U-Net + SE	44.5±10.5	61.5±15.0	27.92±2.23	0.922±0.038	0.932±0.020
3D U-Net + CA	41.2±9.8	58.2±14.2	28.30±2.15	0.927±0.035	0.945±0.018
3D U-Net + CBAM	38.2±5.4 ***	51.0±12.0 ***	29.45±2.10 *	0.940±0.031 ***	0.967±0.015

**Table 3:** Quantitative comparison between the proposed 3D U-Net + CBAM model and representative state-of-the-art sCT generation methods reported in the literature. Results are shown as reported by each study and may not be directly comparable due to differences in datasets and evaluation protocols.

Method	MAE [HU]	PSNR [dB]	SSIM	Dataset/Modality
MU-MedVision (Hybrid CNN–Transformer) [25]	58.83 ± 13.41	29.61 ± 1.79	0.885 ± 0.028	Brain MRI-to-CT
MC-IDDPM [26]	48.83 ± 21.49	26.49 ± 2.81	0.947 ± 0.032	Brain/Prostate MRI
LightGBM Radiomics [27]	60.75 ± 8.8	32.83 ± 2.74	0.88 ± 0.02	Brain (IR-PETRA/VIBE-Dixon)
3D U-Net + CBAM (Proposed)	38.2 ± 5.4	29.45 ± 2.10	0.940 ± 0.0312	Brain MRI-to-CT

## Data Availability

The data supporting the findings of this study are publicly available from the SynthRAD2023 Grand Challenge dataset hosted on Zenodo at https://doi.org/10.5281/zenodo.7260705. To ensure reproducibility and transparency, the full implementation of the proposed CBAM-enhanced 3D U-Net model, including training scripts, inference pipeline, and configuration files, is publicly available on GitHub: https://github.com/shhhsbh/MRI-to-CT-cbam (accessed on 8 January 2026).

## References

[1] Zemmal N., Benzebouchi N.E., Azizi N., Schwab D., Belhaouari S.B. (2022). Unbalanced Learning for Diabetes Diagnosis Based on Enhanced Resampling and Stacking Classifier. Int. J. Intell. Inf. Technol. (IJIIT).

[2] Corradini S., Alongi F., Andratschke N., Belka C., Boldrini L., Cellini F., Debus J., Guckenberger M., Hörner-Rieber J., Lagerwaard F.J. (2019). MR-guidance in clinical reality: Current treatment challenges and future perspectives. Radiat. Oncol..

[3] McRobbie D.W., Moore E.A., Graves M.J., Prince M.R. (2017). MRI from Picture to Proton.

[4] Kurz C., Buizza G., Landry G., Kamp F., Rabe M., Paganelli C., Baroni G., Reiner M., Keall P.J., van den Berg C.A.T. (2020). Medical physics challenges in clinical MR-guided radiotherapy. Radiat. Oncol..

[5] Bahloul M.A., Jabeen S., Benoumhani S., Alsaleh H.A., Belkhatir Z., Al-Wabil A. (2024). Advancements in synthetic CT generation from MRI: A review of techniques, and trends in radiation therapy planning. J. Appl. Clin. Med. Phys..

[6] Nie D., Cao X., Gao Y., Wang L., Shen D. (2016). Estimating CT image from MRI data using 3D fully convolutional networks. Deep Learning in Medical Image Analysis.

[7] Navneet S., Naqvi N.Z. (2023). CT synthesis from MRI using GAN architecture. AIP Conf. Proc..

[8] Liu Y., Chen A., Shi H., Huang S., Zheng W., Liu Z., Zhang Q., Yang X. (2021). CT synthesis from MRI using multi-cycle GAN for head-and-neck radiation therapy. Comput. Med. Imaging Graph..

[9] Pan S., Abouei E., Wynne J., Wang T., Qiu R.L.J., Li Y., Chang C.-W., Peng J., Qiu S., Roper J. (2024). Synthetic CT generation from MRI using 3D diffusion model. Medical Imaging 2024: Image Processing.

[10] Mahalle P.N., Lohani M.C., Hemelatha S., Sharma D., Jayakumar S.S. (2024). Converter Networks for Multi-Scale Tokens-Aware Multi-Region and Multi-Sequence MR-to-CT Synthesis in a Single Model. 2024 Global Conference on Communications and Information Technologies (GCCIT).

[11] Florkow M.C., Zijlstra F., Willemsen K., Maspero M., Berg C.A.T.v., Kerkmeijer L.G.W., Castelein R.M., Weinans H., Viergever M.A., Stralen M.V. (2020). Deep learning–based MR-to-CT synthesis: The influence of varying gradient echo–based MR images as input channels. Magn. Reson. Med..

[12] Oktay O., Schlemper J., Folgoc L.L., Lee M., Heinrich M., Misawa K., Mori K., McDonagh S., Hammerla N.Y., Kainz B. (2018). Attention U-Net: Learning Where to Look for the Pancreas. arXiv.

[13] Ding Z., Zhang Y., Zhu C., Zhang G., Li X., Jiang N., Que Y., Peng Y., Guan X. (2024). CAT-Unet: An enhanced U-Net architecture with coordinate attention and skip-neighborhood attention transformer for medical image segmentation. Inf. Sci..

[14] Azad R., Aghdam E.K., Rauland A., Jia Y., Avval A.H., Bozorgpour A., Karimijafarbigloo S., Cohen J.P., Adeli E., Merhof D. (2024). Medical Image Segmentation Review: The Success of U-Net. IEEE Trans. Pattern Anal. Mach. Intell..

[15] Woo S., Park J., Lee J.-Y., Kweon I.S. CBAM: Convolutional Block Attention Module. Proceedings of the European Conference on Computer Vision (ECCV).

[16] Wang Y., Wang W., Li Y., Jia Y., Xu Y., Ling Y., Ma J. (2024). An attention mechanism module with spatial perception and channel information interaction. Complex Intell. Syst..

[17] Altalib A., McGregor S., Li C., Perelli A. (2025). Synthetic CT image generation from CBCT: A Systematic Review. IEEE Trans. Radiat. Plasma Med. Sci..

[18] Wang J., Yu Z., Luan Z., Ren J., Zhao Y., Yu G. (2022). RDAU-Net: Based on a Residual Convolutional Neural Network with DFP and CBAM for Brain Tumor Segmentation. Front. Oncol..

[19] Zhang B., Qiu S., Liang T. (2024). Dual attention-based 3D U-Net liver segmentation algorithm on CT images. Bioengineering.

[20] Li L., Qin J., Lv L., Cheng M., Wang B., Xia D., Wang S. (2023). ICUnet++: An Inception-CBAM network based on U-Net++ for MR spine image segmentation. Int. J. Mach. Learn. Cybern..

[21] Balaji P., Alsid L.E.G., Mishra S., Obaid A.J., Alkhafaji M.A. (2024). Brain Tumor MRI Segmentation Using Deep Instance Segmentation with Bioinspired Optimization Algorithm. Doctoral Symposium on Computational Intelligence.

[22] Li X., Lan Z., Sun Y., Sun Y., Guo Y., Wang Y., Yuan A. (2025). DPF-Unet: A CNN-swin transformer fusion network for 3D brain tumor segmentation in MRI images. J. Supercomput..

[23] Thummerer A., van der Bijl E., Galapon A.J., Verhoeff J.J.C., Langendijk J.A., Both S., van den Berg C.A.T., Maspero M. (2023). SynthRAD2023 Grand Challenge dataset: Generating synthetic CT for radiotherapy. Med. Phys..

[24] Gul M.S.K., Mukati M.U., Bätz M., Forchhammer S., Keinert J. Light-field view synthesis using a convolutional block attention module. Proceedings of the 2021 IEEE International Conference on Image Processing (ICIP).

[25] Huijben E.M.C., Terpstra M.L., Galapon A.J., Pai S., Thummerer A., Koopmans P., Afonso M., Van Eijnatten M., Gurney-Champion O., Chen Z. (2024). Generating synthetic computed tomography for radiotherapy: SynthRAD2023 challenge report. Med. Image Anal..

[26] Pan S., Abouei E., Wynne J., Chang C., Wang T., Qiu R.L.J., Li Y., Peng J., Roper J., Patel P. (2023). Synthetic CT generation from MRI using 3D transformer-based denoising diffusion model. Med. Phys..

[27] Hoseinipourasl A., Hossein-Zadeh G., Sheikhzadeh P., Arabalibeik H., Alavijeh S.K., Zaidi H., Ay M.R. (2025). Generation of synthetic CT from MRI for MRI-based attenuation correction of brain PET images using radiomics and machine learning. Med. Phys..

